# Semantic segmentation dataset authoring with simplified labels

**DOI:** 10.1007/s11548-024-03314-9

**Published:** 2025-04-05

**Authors:** Leo Uramoto, Yuichiro Hayashi, Masahiro Oda, Takayuki Kitasaka, Kensaku Mori

**Affiliations:** 1 Graduate School of Informatics, Nagoya University, Furo-cho, Chikusa-ku, Nagoya, Aichi 464-8601 Japan; 2 Information Technology Center, Nagoya University, Furo-cho, Chikusa-ku, Nagoya, Aichi 464-8601 Japan; 3 Aichi Institute of Technology, 1247 Yachigusa, Yakusa-cho, Toyota, Aichi 470-0356 Japan; 4 Research Center of Medical Big Data, National Institute of Informatics, 2 Chome-1-2 Hitotsubashi, Chiyoda City, Tokyo 101-0003 Japan

**Keywords:** Semantic segmentation, Dataset authoring, Laparoscopic surgery, Computer vision

## Abstract

**Purpose:**

Semantic segmentation of laparoscopic images is a key problem in surgical scene understanding. Creating ground truth labels for semantic segmentation tasks is time consuming, and in the medical field a need for medical training of annotators adds further complications, leading to reliance on a small pool of experts. Previous research has focused on reducing the time to author datasets, by using spatially weak labels, pseudolabels, and synthetic data. In this paper, we address the difficulties caused by the need for medically trained annotators, hoping to enable non-medical annotators to participate in medical annotation tasks, to ease the creation of large datasets.

**Methods:**

We propose simplified labels, labels that are semantically weak. Our labels allow non-medical annotators to participate in medical dataset authoring, by lowering the need for medical expertise. We simulate authoring processes with mixtures of medical and non-medical annotators and measure the impact adding non-medical annotators has on accuracy. We also show that simplified labels offer a simple formulation for multi-dataset training.

**Results:**

We show that simplified labels are a viable approach to dataset authoring. Including non-medical annotators in the authoring process is beneficial, but medically trained annotators are worth multiple non-medical annotators, with maximal Dice score increases of 9.3% for 1 medically trained annotator and 6.9% for 3 non-medical annotators. We also show that the labels offer a simple formulation for multi-dataset training, even with no overlapping classes. We find that converting the labels of a secondary incompatible dataset into simplified labels and jointly training on both datasets improves performance.

**Conclusion:**

Simplified labels offer a framework that can be applied both to dataset authoring and to multi-dataset training. Using the proposed method, non-medical annotators can participate in semantic segmentation dataset authoring. Labels of incompatible datasets can be converted into simplified datasets, enabling multi-dataset training.

## Introduction

Laparoscopic surgeries offer a minimally invasive option to open surgeries in abdominal and pelvic operations. In a laparoscopic surgery, surgical tools, including a camera system, are inserted into the operating area through small incisions. This approach can lead to faster recovery times, and reduced scarring and pain when compared to open surgery.

Laparoscopic surgeries can be performed with robotic assistance. In addition to providing more intuitive tool control, such robotic systems could offer various kinds of AI-based assistance to the surgical team, varying from guidance systems to automated evaluation and feedback.

Semantic segmentation is a key problem in the development of surgical assistance systems using computer vision, such as intraoperative guidance systems displaying anatomical structures, or safe dissection zones [1]. Surgical semantic segmentation can also act as a base for more complicated problems, like navigation assistance. Semantic segmentation requires a large amount of labeled data, and authoring such datasets is time consuming. A need for medical expertise poses an additional challenge for medical computer vision. Relying on a small pool of expert annotators leads to small datasets, potentially hindering model generalization. To alleviate this, we propose an approach that allows laypeople without medical expertise to participate in the authoring process.

## Related work

There exist only a few large publicly available datasets on semantic segmentation of laparoscopic images. The largest segmentation dataset available at the time of writing is the Dresden Surgical Anatomy Dataset [2], consisting of 13,195 images, and covering 11 classes. However, this dataset does not contain dense annotations for everything in view, but instead contains one class per image. The largest densely labeled dataset we are aware of is the CholecSeg8k [3], containing 8080 images, and covering 13 classes. The frames in CholecSeg8k were sampled at 25 fps, meaning that the whole dataset consists of approximately 5.5 min of video, with at times very low variability. The training portion of the Endoscopic Vision 2018 Robotic Scene Segmentation Challenge dataset [4] (EndoVis2018) dataset consists of 4500 frames and their segmentation ground truths. The frames are sampled at 1 fps from 15 videos of porcine training procedures, performed with the daVinci system. Nephrec9 [5] is a large nephrectomy video dataset, consisting of 1262 clips, each containing 720 frames, for a total of 908,640 frames, each annotated with surgical steps. Instrument and anatomy presences were annotated based on their usage and appearance during steps.

Large datasets are a key to improving performance of machine learning models. Modern, increasingly complicated models are trained on massive amounts of data. One example is the Segment Anything Model [6], trained on over 1 billion masks on 11 million images. In medical imaging, three key areas of research can be identified: semi-supervised training, efficient dataset authoring, and multi-dataset training.

In semi-supervised learning [7], only a portion of the data is labeled. The unlabeled samples have been used as a background for procedurally created tools and their labels in a tool segmentation task [8], with CycleGAN used to make the synthetic samples more realistic. Using 300 annotated samples and 5665 synthetic samples, the authors were able to increase tool segmentation Dice score by 35.7%. Another approach is to create pseudolabels for unlabeled data, effectively extending the dataset during training. Using optical flow for creating pseudolabels [9], researchers were able to train their segmentation models on unlabeled frames as well. Using the labels for only parts of the data, they were able to increase the baseline Dice score by 5.4% when only 10% of the images were labeled, and by 3% when using 30% of the labeled images in a tool segmentation task. On a more complicated part segmentation task, the Dice scores improved by 2.6% with 10% and 3.2% with 30% of the data. When comparing their proposed approach to a model trained on the full training set, their model reached 96% of the performance of the baseline model in part segmentation, and in binary tool segmentation even surpassed it. While semi-supervised approaches have found success in the medical field [10], they do not offer the same level of reliability as fully supervised approaches. This can make them problematic in the medical field, where accuracy is of utmost importance. Training using semi-supervised learning is also sensitive to noise and images dissimilar to the labeled examples, leading to difficulties with creating pseudolabels and to unhelpful synthetic data.

Faster methods to authoring ground truth segmentation maps have been researched. Such labels are typically spatially weaker than dense segmentation maps. Examples of such approaches are bounding boxes [11], scribbles, lines, and points [12]. When comparing the efficacy of their EasyLabels [13] to traditional spatially dense labels, the performance of a model trained on EasyLabels achieved 97% performance on binary tool segmentation, and 90% performance on multiclass segmentation, when compared to a model trained on spatially dense labels. The method offers a significant annotation speed increase, with authors reporting annotation speeds of 428 s per image with normal annotations and 31 s per image with EasyLabels. Scribble annotations were found to take on average only 16 s per slice, box annotations 20 s, point annotations only a few seconds, and full annotations 200 s, when annotating COVID-19 infection lesions in CT images [14]. Class presence labels can be considered an extreme form of spatially weak labels, lacking spatial presence altogether.

The need for large datasets could be alleviated if it was possible to easily train models on multiple datasets. In practice datasets are often incompatible, due to as an example class differences. Multi-dataset training on incompatible datasets has been researched, and the proposed solutions typically use semantic hierarchies [15] and class relationships [16].

While improving both authoring efficiency of medical annotators, and learning from unlabeled data are important, these works overlook an untapped resource in non-medical annotators. We aim to design a segmentation dataset authoring scheme that allows for non-medical annotators to participate in the authoring process, in the hopes of assisting in the creation of larger datasets.

We introduced the preliminary idea of simplified labels in our presentation at CARS 2023 [17]. Simplified labels are a type of label that, in contrast to spatially weak labels, is weak semantically. Instead of reducing authoring time, the goal is to reduce the need for medical training in dataset authoring, allowing non-medical annotators to participate in the process.

In this work, we use the same core methods, but extend the work in multiple areas. We search for an optimal training strategy for the labels, finding a training strategy better than the one proposed in our previous work. We perform a wider range of experiments, adding an additional model to our experiments, showing that the approach is model independent. We also show that the proposed labels offer a simple formulation for multi-dataset training. This approach can be used to include additional training data in a wide variety of surgical image segmentation projects, with minimal effort. We show its feasibility by jointly training on a cholecystectomy dataset, and a porcine dataset, with no overlapping classes. While completely disjoint datasets make for an academically interesting case, differences in annotation granularity in large annotation projects offer a more practical problem solved by this method.

## Methods

When authoring medical segmentation datasets, for problems excluding the simplest ones such as binary tool segmentation, it is necessary for the annotators to possess some level of medical expertise. Here, we define a medical annotator as an annotator capable of identifying all tools and tissue types correctly. In practice, this would correspond to annotators with special training on annotating surgical images, or medical experts. We also define a non-medical annotator as an annotator lacking the training required for annotating surgical images, but having experience in creating segmentation annotations.

Our goal is to build a simplified labeling scheme, in which anon-medical annotator can label basic structures, that can be recognized without medical training. As an example, identifying all different tools would be a difficult task for a non-medical annotator, but one could always recognize that these tools differ from organs and tissue. This information could provide valuable guidance to machine learning models, at a lowered authoring cost in terms of specialist hours. These simple structures can be thought of as groups, containing one or more classes.

Semantic segmentation problems are inherently linked to classification problems. In a classification problem, a single instance is labeled, while in semantic segmentation, each pixel in an input image is given a label. We build our proposed method by first considering a classification problem and then apply it to semantic segmentation.

In classification tasks, categorical variables are often encoded as probability vectors. In a problem with *n* classes, the categorical variable would be encoded as $\textbf{y}$, a nonnegative vector of finite length *n*, such that the sum of the elements of $\textbf{y}$ equals 1.

**Fig. 1:**
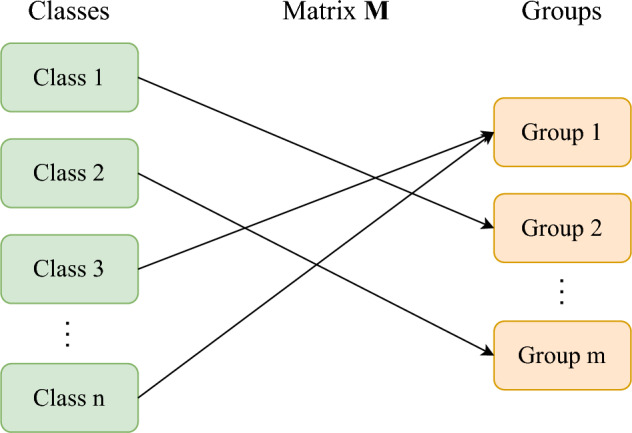
Matrix $\textbf{M}$ encodes the relationships between classes and groups and transforms a probability vector denoting class to a probability vector denoting group

Simplified labels can be generated by grouping classes together. As an example, all tool classes could be collected into a “Tool” group. In a classification task with *n* classes, the classes can be assigned into *m* groups. The relationship between classes and groups, illustrated in Fig. 1, can be described with an $m \times n$ matrix $\textbf{M}$, defined element-wise. The element at index *i*, *j*, $m_{ij}$ is defined as

$$\begin{aligned} m_{ij} = \left\{ \begin{array}{ll} 1 &  \text{ if } \text {class}\,\, \text {j is a member of group } \,\, i, \\ 0 &  \text{ otherwise }. \end{array} \right. \end{aligned}$$

If each class belongs to exactly one class, and if $\textbf{y}$ is a nonnegative vector, then so is the transformed vector $\textbf{M} \textbf{y}$. Furthermore, when defining $\textbf{y}' = \textbf{M} \textbf{y}$, the vectors $\textbf{y}$ and $\textbf{y}'$ have the same element-wise sum,

$$\begin{aligned} \sum _{i=1}^n y_i = \sum _{j=1}^m y'_j. \end{aligned}$$

As a special case, it can be seen that if $\textbf{y}$ is a probability vector, then so is $\textbf{y}'$. The vector $\textbf{y}$ can be interpreted as containing a probability $y_i$ for each class $i=1,\dots ,n$. The simplified vector $\textbf{y}'$ can similarly be interpreted as having a probability for each group $y'_j$ for groups $j=1,\dots ,m$. It can also be seen that if $\textbf{y}$ is a 1-hot encoded vector, then so is $\textbf{y}'$.

Due to these properties, simplified vectors are valid inputs for classification loss functions. Denote a true label with $\textbf{y}$, a predicted probability vector with ${\hat{\textbf{y}}}$, and a classification loss function with *L*. Calculating a loss on the transformed vectors $L(\textbf{M}{\hat{\textbf{y}}},\textbf{M}\textbf{y})$ is a valid operation, calculating the dissimilarity of the vectors on the group level.

**Fig. 2:**
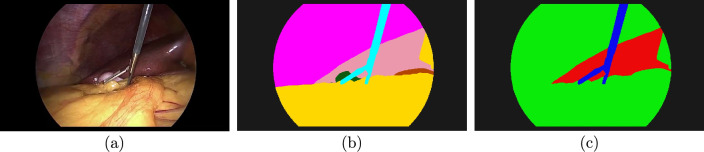
Comparing simplified labels and strong labels. It takes expert knowledge to label everything in the input image (**a**) correctly. A medical annotator can create a strong label (**b**), with grasper (light blue), gallbladder (dark green), liver (pink), gastrointestinal tract (brown), fat (yellow), abdominal wall (magenta), and background (black). A non-medical annotator could create a simplified label, labeling organs (red), tissue (green), tools (blue), and background (black)

This idea can now be transferred to semantic segmentation. A strong label consists of a class-level ground truth for each pixel. A simplified label on the other hand consists of a group-level ground truth label, and a matrix $\textbf{M}$ describing the grouping. The difference between a strong label and a simplified label can be seen in Fig. 2. The strong labels require medical annotators recognizing all classes present in the image, while semantically weaker simplified labels could be authored by non-medical annotators as well. With slight abuse of notation, we skip the tensor notation for segmentation maps and instead use $\textbf{y}$ as a representative of the probability vector of an arbitrary pixel, assuming that each operation is done in a similar way to all pixels. Similarly, the probability vector of an arbitrary pixel in a simplified segmentation map is denoted by $\textbf{y}'$, and in the context of simplified segmentation maps, the simplification process $\textbf{y}'=\textbf{M}\textbf{y}$ is assumed to be performed for each vector in the segmentation map.

The workflow for authoring simplified labels would begin with a non-medical annotator first choosing an appropriate grouping for their skills. As an example, one could author labels with groups “Tissue” and “Non-tissue.” The author would then create dense simplified segmentation maps for input images they are responsible of labeling.

A simplified training sample is a triplet $\{\textbf{x}, \textbf{y}', \textbf{M}\}$, where $\textbf{x}$ is an input image, $\textbf{y}'$ its simplified label and $\textbf{M}$ a matrix built from the grouping the author used. Training on such a triplet is done by predicting a segmentation map ${\hat{\textbf{y}}}$ for the input image $\textbf{x}$, then simplifying the output ${\hat{\textbf{y}}}$ to match the label, and comparing them in a loss function, namely

$$\begin{aligned} \text {Loss} = L(\textbf{M}{\hat{\textbf{y}}}, \textbf{y}'). \end{aligned}$$

When $\textbf{M}=\textbf{I}$, this simplifies to regular supervised training on strong labels.

## Experiments and results

### Overview

To measure usefulness of the proposed simplified labels, we simulated their use in dataset authoring. To simulate a non-medical annotator, first a grouping was chosen, resulting in a transformation matrix. When a training sample was loaded, the ground truth label $\textbf{y}$ was first simplified by transforming it as described in Sect. “Methods” into a simplified label $\textbf{y}'$. The input image $\textbf{x}$, the label $\textbf{y}'$, and the transformation matrix $\textbf{M}$ formed then a training sample $\{\textbf{x}, \textbf{y}', \textbf{M}\}$ that the models were trained on when using simplified data.

We performed three sets of experiments; we first aimed to find the optimal training strategy for the proposed simplified labels, then explored how useful they would be in practice, and finally showed that the proposed approach offers a simple formulation for multi-dataset training. We performed our experiments using the CholecSeg8k [3] dataset in all experiments and used the EndoVis2018 [4] as a secondary dataset in the multi-dataset experiment.

### Model and datasets

The CholecSeg8k dataset contains 8080 endoscopic images with a resolution of $869 \times 512$ pixels, sampled from 17 cholecystectomy videos. Ground truth segmentation labels, covering 13 classes, are provided for all images. Some of the classes are rare, appearing in only few videos. We chose the 8 most common classes and used the 6210 frames consisting only of these 8 classes.

EndoVis2018 consists of porcine training procedures, performed with the daVinci system. The dataset consists of 15 videos, sampled at one frame per second, each containing 300 frames.

We designed an experiment for a training dataset split into four parts. Simulated authors, medical and non-medical annotators, forming teams of 1–4 members, labeled a dataset. Each team member could create labels for only one part of the dataset. Medical annotators created strong labels on the class level, and non-medical annotators simplified labels. The non-medical annotators were able to recognize basic structures, leading to a grouping defined in Table 1. This grouping was chosen due to the groups looking visually distinct from each other. Tools clearly form their own visual group, while organs have shapes and surfaces that make them distinct from the tissue group. As meaningful training requires at least some strong labels, only team compositions with at least one medical annotator were considered.

In all of our experiments, we used fivefold cross-validation, repeating each experiment 5 times for a total of 25 samples. Fourfold was used as the training set, with each fold operated by a simulated author, and onefold as a test set.

We performed our experiments using two models. We chose the DeepLabV3 [18] model for its good performance with even small amounts of data, and the FCN [19] for its simplicity. We used ResNet [20] as the encoder for both models. We trained the models with the Adam [21] optimizer, with a learning rate of 0.0005. Due to class imbalances, we chose Combo Loss [22] as the loss function. Performance was measured with mean Dice score.

Our models were trained using an NVDIA A100 80GB GPU and an AMD EPYC 7402 CPU. The computational time required for one experiment varied from 9 h when using only onefold for training, to 32 h when using the full fourfold of training data available.

### Training strategy

**Table 1:** Classes and groupings in the CholecSeg8k dataset, showing how the simulated medical annotators (class) and non-medical annotators (group) labeled the dataset

Class	Group
Abdominal wall	Tissue
Fat	Tissue
Gastrointestinal tract	Tissue
Liver	Organ
Gallbladder	Organ
Grasper	Tool
L-Hook	Tool
Background	Background

**Table 2:** The three training strategies compared

	Stage 1			Stage 2			Stage 3		
	Data type	lr	Epochs	Data type	lr	Epochs	Data type	lr	Epochs
Strong first	Strong	0.0005	10	Simplified	0.0005	10	Strong	0.000125	5
Simple first	Simplified	0.0005	10	Strong	0.0005	10	Strong	0.000125	5
Mixed	Mixed	0.0005	10	Mixed	0.0005	10	Strong	0.000125	5

Data type shows the type of data used in a stage, lr the learning rate, and epochs the length of the stage. When mixed data is used, 5 epochs of both strong and simplified data are interleaved for a total of 10 epochs

Due to size mismatches, simplified and strong labels cannot be trained on in the same batch. Strong labels are tensors of size $H \times W \times n$, where *H* and *W* are the width and height of the image, and *n* the number of classes. Simplified labels on the other hand are of size $H \times W \times m$, where *m* is the number of groups. Due to the size mismatch, these tensors cannot exist in the same batch. As sampling the dataset in minibatches was not possible, it was necessary to create a training strategy for the simplified labels.

We trained our model in 3 phases, consisting of 10, 10, and 5 epochs. In the third phase, we lowered the learning rate from the initial 0.0005 to 0.000125. With this setting, 3 training strategies were considered: training first on the strong data, training first on the simplified data, and using a mixed approach. These training strategies are visualized in Table 2.

#### Strong first

In strong first training, the model was first trained on the strongly labeled data for 10 epochs, then for 10 epochs on the simplified data, and finally for 5 epochs on the strongly labeled data with a lowered learning rate. We hypothesized that training first on the strongly labeled data might allow the model to extract more meaningful information from the semantically weak labels.

#### Simplified first

In simplified first training, the model was first trained for 10 epochs on data with simplified labels, followed by 15 epochs on strongly labeled data, with the learning rate lowered for the last 5 epochs. This approach could be thought of as pretraining the model on the simplified data, in preparation for training on the strongly labeled data. Pretraining is often used in practice, which suggested this could have been a good approach.

#### Mixed

In a mixed approach, the simplified data and the strongly labeled data were interleaved for a total of 20 epochs, attempting to mimic minibatch training. After training the model on strongly labeled data for one epoch, the model was trained for one epoch on data with simplified labels. After these 20 epochs, the model was trained for 5 epochs on strongly labeled data with a lowered learning rate.

#### Experiment and results

**Table 3:** Results from the training strategy experiment with the DeepLabV3 and FCN models

	DeepLabV3			FCN		
	1 M, 3 NM	2 M, 2 NM	3 M, 1 NM	1 M, 3 NM	2 M, 2 NM	3 M, 1 NM
Simplified first	$0.696\pm 0.042$	$0.753 \pm 0.047$	$0.804 \pm 0.039$	$0.701\pm 0.058$	$0.760 \pm 0.040$	$0.805 \pm 0.035$
Strong first	$0.722 \pm 0.048$	$0.758 \pm 0.045$	$0.808 \pm 0.041$	$0.721 \pm 0.046$	$0.765 \pm 0.044$	$0.794 \pm 0.038$
Mix	$\mathbf {0.736} \pm 0.042$	$\mathbf {0.772}\pm 0.043$	$\mathbf {0.821}\pm 0.032$	$\mathbf {0.730} \pm 0.042$	$\mathbf {0.772}\pm 0.040$	$\mathbf {0.810}\pm 0.037$

The number of medical annotators is denoted with M, and the number of non-medical annotators with NM

For each column, the highest value is bolded

For the training strategy experiment, we used 3 representative team compositions: 1 medical and 3 non-medical annotators, 2 medical and 2 non-medical annotators, and 3 medical and 1 non-medical annotators. These samples covered imbalances favoring both strongly labeled and simplified data and a balanced sample. The results of the experiments are collected in Table 3.

**Fig. 3:**
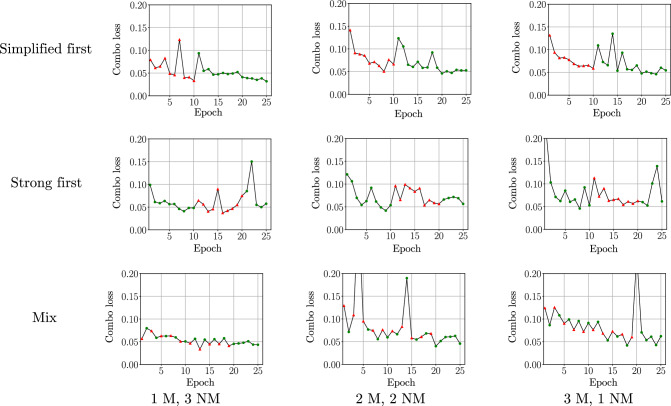
Learning curves showing training losses of the training strategy experiment. The number of medical annotators is denoted with M and non-medical annotators with NM. Epochs with simplified labels are marked with red triangles, and epochs with strong labels with green dots

The results showed that the mixed strategy performed the best and was adopted as the training strategy for the other experiments.

To verify that the training had converged, we looked at learning curves, shown in Fig. 3, from our experiments with the FCN model. The losses seemed to converge reasonably well with all of the training strategies, settling near Combo Loss value 0.05.

**Fig. 4:**
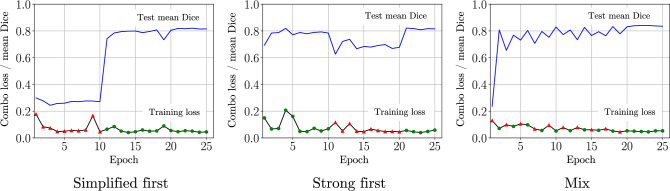
Learning curves of the training strategy experiment with two medical and two non-medical annotators, showing also performance on the test set. The mean Dice on the test set is shown in blue, and the training loss in black. For training data, epochs with simplified labels are marked with red triangles, and epochs with strong labels with green dots

Seeking to better understand the differences between the training strategies, we performed an additional training run on 2 simplified and 2 strong folds, tracking the model’s performance on the test set through training. We chose FCN for this experiment as it was the simpler model. Figure 4 shows the mean Dice on the test set at the end of each iteration. In general, the performance on the test set falls immediately after an iteration on simplified data, but after training again on strongly labeled data, the performance immediately improves. With the mixed strategy, performance degrades less and less after epochs on simplified data, while performance after epochs on strongly labeled data improves. On the last 5 epochs, when using a lowered learning rate, the model trained with the mixed strategy learns to perform the best on the test set, at a higher loss value than the other two. At the end of the training, its final mean Dice value is 0.835, significantly higher than the maximum mean Dice scores of the simplified first (0.821) and strong first (0.822) over the whole training phase.

### Measuring the impact of simplified labels

With the selected mixed training strategy, we explored the impact of the simplified data on performance, by collecting performance metrics for all 10 team compositions in Table 4 for FCN and Table 5 for DeepLabV3. Including simplified data was always useful for DeepLabV3, even when the amount of simplified data outweighed the strongly labeled data by a 3:1 ratio. For FCN, we found small amounts of data detrimental, with rest of the results agreeing with the trend of DeepLabV3 data. It is difficult to explain this result, but changing the number of epochs or the learning rate when using small amounts of simplified data could lead to better results with this model. For both models, even large amounts of simplified data could not replace strongly labeled data; one medical annotator was worth multiple non-medical annotators.

**Table 4:** Full results of the main experiment for the FCN model

	1 M	2 M	3 M	4 M
0 NM	$0.703 \pm 0.058$	$0.764 \pm 0.041$	$0.812 \pm 0.031$	$0.839 \pm 0.026$
1 NM	$0.697\pm 0.043$	$0.760 \pm 0.034$	$0.809 \pm 0.028$	–
2 NM	$0.718\pm 0.040$	$0.772 \pm 0.042$	–	–
3 NM	$0.736\pm 0.041$	–	–	–

The number of medical annotators is denoted with M, and the number of non-medical annotators with NM

**Table 5:** Full results of the main experiment for the DeepLabV3 model

	1 M	2 M	3 M	4 M
0 NM	$0.696 \pm 0.064$	$0.761 \pm 0.044$	$0.813 \pm 0.038$	$0.847 \pm 0.030$
1 NM	$0.701\pm 0.043$	$0.765 \pm 0.044$	$0.821 \pm 0.034$	–
2 NM	$0.729\pm 0.046$	$0.774 \pm 0.042$	–	–
3 NM	$0.737\pm 0.039$	–	–	–

The number of medical annotators is denoted with M, and the number of non-medical annotators with NM

**Fig. 5:**
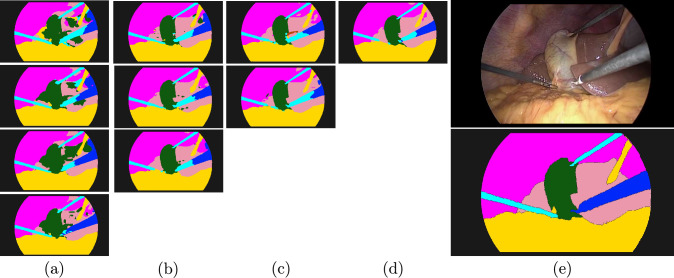
A qualitative comparison with DeepLabV3. The input image and ground truth **e** show two graspers (light blue), and an electrocautery tool (dark blue) operating on a gallbladder (green), next to a liver (pink), and abdominal wall (magenta). Columns show 1 medical annotator and 0–3 non-medical annotators (**a**), 2 medical annotators and 0–2 non-medical annotators (**b**), 3 medical annotators and 0–1 non-medical annotators (**c**), and 4 medical annotators and 0 non-medical annotators (**d**)

Qualitatively, the impact of simplified data on DeepLabV3 can be seen in Fig. 5. All models segment the fat well, but with other classes results vary. Impact of the simplified labels can be seen most clearly on tools, where shapes are improved. With one medical annotator, the electrocautery tool improves significantly. The best result for the tool being with one medical and three non-medical annotators is most likely caused by the model seeing less strongly labeled data where only tool shafts were visible, which would have led to the model learning potentially unhelpful features. In general, adding simplified data led to more local consistency with a lack of noisy speckles, and improved shapes, especially on the right side of the image.

We concluded that the proposed simplified labels are a feasible approach to dataset authoring when the process is limited by access to medical annotators. Non-medical annotators provide meaningful assistance to dataset authoring.

#### Comparing the impacts of medical and non-medical annotator labels

Data found in Tables 4 and 5 can be used to compare the impacts strong and simplified labels have on performance. We measured the statistical significance of the performance changes using two-tailed paired Student’s t test. With one medical annotator, adding three non-medical annotators to the project resulted in mean Dice score increase of 5.8% for DeepLabV3 ($p<.001$), and 4.7% for FCN ($p=.035$), while adding one medical annotator resulted in an increase of 9.2% for DeepLabV3 ($p<.001$) and 8.6% for FCN ($p<.001$). With two medical annotators, adding two non-medical annotators resulted in an increase of 1.8% for DeepLabV3 ($p=.004$) and 1% for FCN ($p=.149$), while adding one medical annotator resulted in 6.9% ($p<.001$) and 6.3% ($p<.001$) increases for DeepLabV3 and FCN, respectively.

From these results, it can be seen that one medical annotator is worth multiple non-medical annotators.

### Simplifying other datasets

The problem of small datasets could be combated by training on multiple datasets. However, in practice datasets are often incompatible, containing different classes. Simplified labels could be used to solve such incompatibilities, using a grouping that makes data from both datasets compatible.

We performed an experiment with an extreme example, using the EndoVis2018 dataset as the secondary dataset, aiming to improve the performance of our CholecSeg8k-targeted model. EndoVis2018 has a resolution of 1280 $\times$ 1024 pixels, making the images significantly larger than those of CholecSeg8k. To keep feature sizes on both datasets similar, EndoVis2018 images were scaled down to a resolution of 640 $\times$ 512 pixels. EndoVis2018 contains 8 classes for tools: 3 classes for parts of the daVinci system, ultrasound probes, suturing needles, suturing thread, suction devices, and surgical clips. There are 4 different types of tissue: kidney parenchyma, covered kidney, small intestine, and other tissue considered background. There is no meaningful overlap with the CholecSeg8k dataset; the EndoVis2018 tools and organs are different, and the subject is non-human. We simplified the classes in both datasets to “biological,” “non-biological,” and “CholecSeg8k background” and applied the simplified label workflow.

We trained both DeepLabV3 and FCN models on the 4 medical annotator folds of the CholecSeg8k dataset, included EndoVis2018 as simplified labels, and measured the performance on the remaining CholecSeg8k fold. We compared these results against the 4 medical annotator data from Tables 4 and 5 and collected the data in Table 6.

**Table 6:** Mean Dice scores when training on 4 strongly labeled folds, with and without a simplified EndoVis2018 dataset

Dataset	CholecSeg8k	CholecSeg8k + EndoVis2018
DeepLabV3	$0.847 \pm 0.030$	$\mathbf {0.855} \pm 0.030$
FCN	$0.839 \pm 0.026$	$\mathbf {0.845} \pm 0.028$

For both rows, the highest value is bolded

**Table 7:** Dice scores for each class when training a DeepLabV3 model on 4 strongly labeled folds, with and without a simplified EndoVis2018 dataset

Dataset	CholecSeg8k	CholecSeg8k + EndoVis2018
Abdominal wall	$0.928 \pm 0.036$	$\mathbf {0.930} \pm 0.032$
Fat	$0.916 \pm 0.031$	$\mathbf {0.919} \pm 0.030$
Gastrointestinal tract	$\mathbf {0.634} \pm 0.169$	$0.625 \pm 0.190$
Liver	$0.918 \pm 0.020$	$\mathbf {0.920}\pm 0.019$
Gallbladder	$0.830 \pm 0.068$	$\mathbf {0.854}\pm 0.049$
Grasper	$0.823 \pm 0.041$	$\mathbf {0.848}\pm 0.029$
L-Hook	$0.735 \pm 0.272$	$\mathbf {0.752} \pm 0.254$
Background	$0.993 \pm 0.003$	$\mathbf {0.994}\pm 0.002$

For each row, the highest value is bolded

Table 6 shows that both models benefited from additional data. It also shows that the DeepLabV3 model had a higher mean Dice value both with and without the secondary dataset, and it benefited more from the inclusion of the secondary dataset. As the better performing model, we selected DeepLabV3 for further analysis. We collected Dice scores of all classes to explore the impact of training on the secondary dataset and collected the data in Table 7 to see which classes benefited from the secondary dataset. Table 7 shows that including the EndoVis2018 dataset resulted in performance gains in almost all classes with the DeepLabV3 model, with the exception of gastrointestinal tract. The mean Dice improved by approximately 1% with both models.

We concluded that the proposed approach could be used to train on multiple incompatible datasets.

## Discussion

Massive datasets have been a trend not only with language models, but also with recent segmentation models like the Segment Anything Model. The idea of creating similar large models in areas like semantic segmentation of laparoscopic images is challenging, as massive datasets are significantly more difficult to author. Creating one large dataset once would not suffice, as it could become outdated due to new technologies in cameras and tools.

Improving the efficiency of medical annotators is an important step toward larger datasets, but involving non-medical annotators in the authoring process could have its benefits. Creating large datasets covering a wide variety of different images could be realized by crowdsourcing help from non-medical annotators around the world. In annotation projects with a limited number of medical annotators, or projects with time limitations, non-medical annotator participation could help increase the number of annotated frames and help with achieving desired levels of accuracy. Simplified labels could be used in such situations.

**Table 8:** Dice scores of gastrointestinal tract (GI tract) and L-hook from the CholecSeg8k 10 team experiment with DeepLabV3

	1 M	1 M 1NM	1 M 2NM	1 M 3NM	2 M	2 M 1NM	2 M 2NM	3 M	3 M 1NM	4 M
GI tract	0.241	0.163	0.205	0.214	0.294	0.285	0.273	0.514	0.510	0.634
L-hook	0.598	0.609	0.624	0.618	0.595	0.621	0.649	0.677	0.703	0.734

The number of medical annotators is denoted with M, and the number of non-medical annotators with NM

The granularity of the simplified labels can be matched to the skill level of the author. We showed that even the weakest labels, consisting of groups “biological” and “non-biological,” with input images extracted from a different distribution, helped with training.

Semi-supervised approaches where data are created at training time can be challenging for quality control. Confirming the correctness of simplified labels on the other hand is easy, making the approach potentially more suitable for medical applications. We do want to emphasize that simplified labels do not have to fully replace semi-supervised approaches or directly compete with them, as it is possible to both use the simplified labels approach to authoring a labeled dataset, and to use semi-supervised methods like synthetic data and pseudolabels on the remaining unlabeled data.

In all of our experiments, we found the Tool group benefiting the most from simplified labels. This might be due to tools differing the most from other classes, and even in semantically weak form offering meaningful supervision. Gastrointestinal tract stood out with its performance degrading as simplified data were added. Table 8 shows the Dice score L-hook improving, while the Dice score of gastrointestinal tract degraded, when simplified data were included in the training. In Table 7, it can be seen that the gastrointestinal tract was the only class to suffer from simplifying the EndoVis2018 dataset.

The poor performance of the gastrointestinal tract was difficult to explain. Rarity alone did not explain this finding, as the similarly rare L-hook improved with the addition of simplified data. The class appears to simply be difficult to learn, with performance degrading due to simplified data offering only weak supervision for the class.

If multiple difficult-to-learn classes were present, simplified labels might degrade the performance of all of them, making the labels counterproductive. As it is possible to imagine situations where simplified labels could offer no help, they should not to be considered a universal approach to dataset authoring. Even if simplified labels were not a universal approach, our simulations demonstrated their benefits, making them worth considering when designing a dataset authoring project.

Even very similar semantic segmentation datasets pose problems for joint training, by as an example containing different classes, or having different granularities. Authors of a dataset might have focused on specific organs or tools, worked at a granularity not desired by someone else, or have different tools in their images. Being able to include such incompatible datasets in a training pipeline is desirable. The multi-dataset training framework we have presented is easy to implement, and can be used with a multitude of different models and training pipelines. We have shown that this approach works even when there are no overlapping classes.

## Conclusions and future work

In this paper, we introduced simplified labels. Unlike spatially weak labels seen in the literature, our labels are weak in their semantic contents. Instead of focusing on authoring speed, they offer a novel way of approaching the dataset authoring problem by allowing for authors with varying degrees of medical expertise to participate in authoring of medical segmentation datasets.

Due to tensor size incompatibilities, simplified and strong labels need to be trained on separately. We compared three possible training strategies, finding that a mixed strategy interleaving training on strong and simplified data worked the best. We explored the impact of simplified labels and found that they improved the performance of our models, but were unable to replace expert annotators completely. We also showed that simplified labels offer a simple formulation to multi-dataset training and found that including a secondary dataset as simplified data improved the performance of our models increased by 1%.

One issue not addressed by this study is the accuracy of the non-medical annotator labels. The authoring process was simulated from pre-existing medical annotator labels, and their accuracy could have been overestimated. It is possible for the non-medical annotators to make errors. In practice, this issue could be solved by having the non-medical annotators label only easy images. Still, measuring the accuracy of real non-medical annotators is worth exploring in future research.
